# Brain Injury due to Mechanical Trauma and Ischemic-Hypoxic Insult: Biomarkers of Brain Injury and Oxidative Stress

**DOI:** 10.1155/2017/8923472

**Published:** 2017-09-24

**Authors:** Margherita Neri, Andreas Büttner, Vittorio Fineschi

**Affiliations:** ^1^Department of Morphology, Surgery and Experimental Medicine, University of Ferrara, Via Fossato di Mortara, 70 44100 Ferrara, Italy; ^2^Institute of Forensic Medicine, Rostock University Medical Center, Rostock, Germany; ^3^Department of Anatomical, Histological, Forensic and Orthopaedic Sciences (SAIMLAL), Sapienza University of Rome, Viale Regina Elena 336, 00185 Rome, Italy

Brain injury (BI) due to mechanical trauma represents one of the major causes of mortality and disability in the world. The pathophysiology of BI is composed of two different pathogenetic moments: primary damage due directly to trauma, with mechanical forces applied to the head, and subsequent biochemical events due to a complex cell response that leads to the death of neuronal cells and classified as secondary damage. Accumulating evidence suggests that the extent of brain injury and the clinical outcome after BI are modulated, to some degree, by genetic variants. In the literature, the existence of a rather precise chronology of expression of the different markers of hypoxic-ischemic brain damage has been shown, which is correlated to the duration of the same insult and is to be ascribed, essentially, to a different stimulation of the different cellular types and also to a different response by the same cells to the ischemic insult.

In brain injury cases, there is a cross-talking network of cellular processes that involves the combination of the two major cell deaths: necrosis and apoptosis. Apoptosis plays an important role in brain development, as immature cells tend to apoptosis even in physiological conditions. In the face of a hypoxic-ischemic injury, apoptosis and necrosis overlap so much so that today it is normal to consider it a continuum of apoptosis-necrosis in a certain injured brain area.

As noted by J. I. Romero et al., it is apparent from the literature that various neuroprotective agents, described as effective from previous studies, have been used to improve the damage caused by hypoxia/ischemia in the central nervous system (CNS); however, none of them have been shown to be effective against damage induced by hypoxia/ischemia in the CNS. Their experimental paper aims to demonstrate the neuroprotective effects of Grx2 and Trx1 expressed exogenously, recombinantly in an animal model of neonatal hypoxia/ischemia, which was designed to look for potential new therapeutic strategies. By performing an elegant experimental procedure, the authors used a common carotid artery binding model in the P7 Sprague-Dawley P7 male rats subjected to a 100% nitrogen environment to induce anoxia. Grx2 and Trx1 were administered to examine the role of Grx2 and Trx1 recombinant as therapeutics after perinatal asphyxiation. The results are encouraging because the authors suggest that Grx2 administration first and Trx1 administration, in a small way, have the potential to significantly mitigate the neuronal damage caused by PA, including cell damage response, glutamate excitotoxicity, axonal integrity, and astrogliosis.

The therapeutic potential of another substance, SCM-198, was used to protect against ischemia-reperfusion injury and possible underlying mechanisms as studied by Q.-Y. Zhang et al. experimentally in rats by inhibition of transient cerebral artery in vivo (tMCAO).

The authors have shown that SCM-198 significantly decreased the volume of infarction and improved the neurologic deficit in the tMCAO model. In addition, SCM-198 could also reduce cellular lesion caused by OGD/R in vitro. Further studies on the mechanism have shown that HDAC4 could inhibit the expression of NOX4 and MMP9 and thus improve TJ levels and protect against blood-brain barrier (BBB) breakdown. This study therefore revealed that HDAC4 was involved in regulating BBB's integrity and that SCM-198 had protective effects against BBB destruction by improving HDAC4 expression. In the genesis of delayed permanent damage to the central nervous system, a primary role is due to oxidative stress. Among the major sources of reactive oxygen species (ROS) in the brain, one should remember that NADPH oxidases (Nox) and ubiquitary membrane multisubunit enzymes are involved in many neurological degenerative diseases. NOX isoforms have been examined as fundamental in the context of TBI-induced NLRP3 inflammasome pathway activation. Recent literature evidence shows that Nox-2 is upregulated after TBI; this objective finding therefore assumes a critical role both at the initial stage and in the subsequent development of this pathology.

M. W. Ma et al. organized an experiment with Nox-2 KO mouse: mice were anesthetized with isoflurane and subjected to an injury or controlled cortical impact. The study has shown that oxidative stress derived from NOX2 induces TXNIP interaction with NLRP3 to induce inflammation of NLRP3, which enhances inflammation in the wounded cortex after TBI. From this study, it is concluded that inflammation of NLRP3 may be useful as an effective treatment for TBI. NLRP3 may also be useful in other acute and chronic cerebral lesions involving inflammation of NLRP3.

A model of lesion of the spinal cord reperfusion (SCIR) was created by L. Xie et al. to evaluate the role of autophagy and its potential signaling path in SCIR and protective effect and internal mechanism of NAD+ involved in SCIR and to explore the underlying relationship between autophagy and NAD+. In conclusion, this study suggests excessive and sustained autophagic activation in SCIR. NAD+ administration can reduce the damage caused by I/R and inhibit neuronal cell apoptosis.

Another fundamental field of research is the neuropathological characteristics of blast polytrauma, a general aspect to better understand the body's response and reflexes on cerebral pathophysiology. In this regard, an interesting line of research has been put in place to document the effects of blast polytrauma with an animal model that also investigates the behavioral response, hypoxic effects, and the consequences of hypoxia itself on brain cellularity. In W. B. Hubbard et al.'s study, by studying reactive astrocytic response (GFAP), apoptosis, cleaved caspase-3 expression, and microglial activation, both in traumatic brain injury and in mild brain injury, and by comparing with the control group, we can explain the organism responses. In addition, by analyzing the integrity of the BBB and hypoxic and angiogenesis expressions, a comprehensive view is obtained to identify concrete and useful polytraumatic-specific injury markers that may be of practical and immediate utility in clinical routine.

The practical development of this experimental model is to examine the role of oxidative stress in a pathophysiological system governed by hypoxia.

Oxidative stress and aberrant deimination are part of the pathology that accompany brain damage. Blast traumatic brain injury causes protein deimination, and this is a reversible process. Basically, by activating calcium-dependent enzymes, aberrant protein deimination is caused causally by traumatic brain injury, in which the pathological response of the adaptive immune system is still largely the case.

In summary, the effects of brain injury due to mechanical trauma represent one of the major causes of mortality and disability in the world. A review of studies from the literature indicates that cell responses and pathological consequences seem to be more or less the same in most animal models, but the models diverge widely when it comes to their ability to produce the changes induced by combined effects due to trauma and/or hypoxia/ischemia in the CNS. The epidemiological importance of the underlying disease makes a thorough knowledge of its direct and indirect traumatic effects fundamental from the research, pathological, and emergency therapy points of view. This special issue wants to contribute for a better understanding on brain injury due to mechanical trauma-related pathophysiology, cellular responses, behavioral response, hypoxic effects, and the consequences of hypoxia itself on brain cellularity.

*Margherita Neri* *Andreas Büttner* *Vittorio Fineschi*

